# Broncho-Pneumonia

**Published:** 1906-05-26

**Authors:** 


### BRONCHO-PNEUMONIA.

Not many years ago the current teaching of our schools was to the effect that in childhood pneumonic affections were invariably lobular in their character: that is to say that fibrinous or lobar pneumonia was at this period of life an extreme rarity, and broncho-pneumonia a practical monopolist of acute lung affections in early life. It is generally recognised now that lobar pneumonia occurs with great frequency in childhood, but its fatality at this age is far smaller than it becomes during adult life. On the other hand, broncho-pneumonia, a lesion of great gravity whatever the age of its victim, is undoubtedly a malady peculiarly associated with early years, and one very largely responsible for the long death-roll of measles and whooping-cough.

It is customary to divide broncho-pneumonic inflammations for purposes of clinical description into three main varieties. Of these two are admittedly secondary to other lesions, while the third, and least important—as being the least frequent—appears to be a primary affection similar to lobar pneumonia, but lobular in its distribution. The two varieties of secondary broncho-pneumonia are: (1) The ordinary form met with most often either as a sequel of a protracted and severe bronchitis; (2) the septic form, called also "inhalation pneumonia," and associated, as its name implies, with the aspiration of infective particles.

By far the larger number of all cases of broncho-pneumonia fall into the former of these two categories. The morbid associations of this variety differ according to the age of the patient, but present certain fundamental similarities, the chief of which is the presence of a bronchitis of the smaller air-tubes. In very young children this bronchitis is often an apparently primary condition, but its victims are almost always children in a poor state of bodily health, either rickety, or debilitated by faulty feeding and the resulting gastro-intestinal disturbances. In older children—let us say between the ages of two and five years—broncho-pneumonia is a rarity except in association with one or other of the two specific fevers whose characteristics include severe bronchitis—namely, measles and whooping-cough. The actual exciting cause of the disease is undoubtedly a micro-organism acting locally, but many varieties of organism appear to be efficient causes of the lesion. The commonest by far is the pneumococcus, either alone or in combination with a variety of other forms, whether streptococci, staphylococci, or, as occasionally happens, the pneumo-bacillus of Friedländer. The morbid appearances vary with the duration of the lesion. In an early case the lung may appear normal to the eye, and yet under the microscope show an intense peri-bronchial infiltration with leucocytes. In a typical case of some standing the uncut lungs show on their serous surfaces alternating areas of air-containing lung and depressed areas of a darker colour indistinguishable to the eye from the appearances of simple lung collapse. There may at the same time be evidence of pleurisy, indicated by the presence of a fibrinous deposit or by simple roughening and injection of the membrane with a slight degree of adhesion between the two surfaces of the pleura. When the lungs are cut across the typical picture of broncho-pneumonia is found to consist of a multitude of pale dots irregularly distributed about the lung and coalescent to a variable degree. These pale areas are somewhat raised above the average level of the cut surface, and are interspersed with depressed zones of a darker colour, representing air-containing lung in varying stages of collapse. The lesion is bilateral, and may, by extensive coalescence of its individual units, present a wide area of consolidation. In very advanced and chronic cases each of the pale areas may be found to be a minute abscess containing a drop of liquid pus. The microscopical characters of a broncho-pneumonic lung in an early stage of the disease are very typical. The outstanding feature of a section of such a lung is a patchily-distributed infiltration of small round cells. The bulk of the field may present alveoli in what is practically a normal condition, although some tendency towards collapse is generally to be detected. Closer examination will show that the areas of cell infiltration are uniformly centred round air-tubes of tolerable size. In the centre of such a patch it is often possible to see a bronchus, easily recognised by its tall ciliated lining epithelium. This epithelium is generally more or less desquamated into the lumen of the tube, where it lies intermingled with a considerable quantity of other cellular debris. The neighbourhood of such a bronchus is densely packed with inflammatory leucocytes, the whole giving a very clear picture of an inflammation spreading by way of the air-tubes. If now attention be directed to a section of lung showing the advanced lesions of the malady it will be found that the whole field is solid with inflammatory exudates. Here and there may be seen a bronchus unnaturally dilated and totally devoid of its lining epithelium. Its lumen is completely occluded by a cellular plug consisting of epithelial cells, connective-tissue cells, and leucocytes. The outlines of the alveoli are generally to be distinguished, but their cavities are filled by desquamated endothelial cells and leucocytes in varying abundance.

It is easy to reconstruct from these two pictures the probable sequence of events which has led to the development of the second from the first. A bacterial invasion of the larger air tubes leads to a bronchial inflammation resulting in desquamation of the lining epithelium and in leucocytic infiltration of the peri-bronchial zone. The lumen of the bronchus becomes plugged, and the area of lung dependent upon it consequently collapsed. The bronchus concerned is thus converted into the analogue of a culture-tube filled with a semi-liquid organic medium and actively pathogenic micro-organisms. The inflammation, aided no doubt by gravity, spreads downwards, and ultimately involves the whole area of lung served by the affected bronchus, the whole process being somewhat analogous to that of infarction after arterial embolism.

The symptoms of such a secondary broncho-pneumonia are vague enough. In a typical case the urgency of the original malady, whether it be a simple bronchitis or a definite specific fever, may appear to decline. That is to say, the fever may fall, or become less persistent, while yet the general appearance of illness is maintained, and the respiratory symptoms remain unmitigated or are actually increased. Cough may diminish although the lesion is becoming more pronounced, and with the increasing debility of the patient may almost disappear. Auscultation of the lungs during the establishment of broncho-pneumonia may show a gradual diminution in the frequency of the râles of bronchitis, with the substitution of smaller râles suggestive of invasion of smaller air-tubes. This is particularly noticeable in the case of broncho-pneumonia secondary to whooping-cough. At times the urgency of the dyspnœa and the gravity of the constitutional disturbance may render the diagnosis of broncho-pneumonia almost a certainty while the physical examination of the chest may reveal nothing but perhaps a slight exaggeration of the normal breath sounds. It is important to recognise that the physical signs typical of broncho-pneumonia—namely, a patchy dullness with a similar distribution of bronchial breathing, are not to be found except in advanced cases, and even then not invariably. Physical considerations will make it apparent that these signs can only occur when gross consolidation takes place in the neighbourhood of bronchi of considerable size. The inflammation may be widespread and grave without conforming to these essentials.

The course of the disease is chronic, as might be assumed from the character of the lesion. Health is seldom regained in less than six or eight weeks, but convalescence may be much more protracted. As the chronic nature of the malady declares itself it becomes a problem of extreme anxiety, whether or not the basis of the lesion is a tubercular process. It may be said at once that there is no positive criterion whereby a simple is to be distinguished from a tuberculous broncho-pneumonia: more than this, there are no guides of even approximate reliability. If the child is known to be the subject of some other tuberculous affection the probabilities in favour of tuberculosis are of course proportionately increased; but this apart, it can only be said that if the process be tuberculous the downward progress of the case is more likely to be rapid than if it is not.

The treatment of broncho-pneumonia, when once it is established, is extremely unsatisfactory. There is no doubt that our efforts ought to be concentrated upon prophylaxis. The disproportionate incidence of broncho-pneumonia upon the children of the poor as compared with those in better circumstances points to the importance of hygienic considerations in the prevention of this disease. There can be little doubt that of all remotely predisposing causes of the disease, deficient ventilation and its attendant evils take the first place. Better housing and some elementary knowledge of hygiene are therefore cardinal principles in the matter, and for these we must live in hope. A consideration of the mechanical laws which govern the motion of exudations in the air-tubes makes it reasonable to suggest that for young children, whose chest-walls and expiratory movements are equally feeble, the benefit of gravity should be commanded by elevating the foot of the bed in the case of such a child affected with anything like a copious bronchitis. Of drugs, belladonna remains a favourite, and its use is based upon rational grounds. But drugs are of secondary importance. The experience of special hospitals is to the effect that of all remedies fresh air is the first in importance; and this even in cases apparently hopeless. It is well to anticipate the almost certain opposition of parents to such a scheme of treatment, but its wisdom justifies insistence upon the matter.

The other variety of secondary broncho-pneumonia, namely, "septic" or "inhalation" pneumonia, has no especial affinity for youth. It is frankly a disease due to the entrance into the air-tubes of infective particles whose origin can generally be detected easily. Thus it may result from any lesion of the upper air-passages which is associated with the production of infective exudates, whether primarily infective, as in the case of diphtheria, or secondarily, as in the case of inflamed laryngeal neoplasms. In either case an established septic broncho-pneumonia is a matter of the greatest possible gravity, and one whose prevention demands the most urgent care. In adults such prevention is too often beyond our reach, since the commonest cause at this period of life is the ulceration of malignant disease into the trachea. In children the commonest cause is probably to be found in a tracheotomy wound. It must be remembered that at all ages an occasional cause is the inhalation of vomited material during unconsciousness, an accident particularly associated with anæsthesia for surgical purposes. It is not necessary to labour the means at hand for preventing this disease in so far as it can be prevented. They are mechanical and obvious. It is permissible, however, to express surprise that, in the after-care of tracheotomy cases, advantage is not more often taken of the simple expedient of raising the foot of the bed in order to prevent the gravitation of discharges down the trachea.

Acute primary broncho-pneumonia does not require detailed notice, for it is a rare malady in this country, although common enough in America, according to Holt. It attacks young children, and is characterised by the sudden and urgent symptoms of lobar pneumonia, but the fever is neither so high nor so persistent as is that of lobar pneumonia, nor does it as a rule terminate by crisis. The disease lasts for two or three weeks.

